# Neuroprotective Effects of Sorghum Polyphenol in Alzheimer’s Disease: In Vitro and In Silico Analyses

**DOI:** 10.3390/nu18132121

**Published:** 2026-06-30

**Authors:** Rasheed A. Abdulraheem, Ralph N. Martins, Rajapandiyan Krishnamoorthy, Mohammad A. Alshuniaber, Prashant Bharadwaj, Zhaoyu Li, Ranil Coorey, Vijay Jayasena, Stuart K. Johnson, W.M.A.D. Binosha Fernando

**Affiliations:** 1 School of Medical and Health Sciences, Edith Cowan University, Joondalup, Perth, WA 6027, Australia; rasheeda@our.ecu.edu.au (R.A.A.); ralph.martins@uwa.edu.au (R.N.M.); bharadwaj.prashant@uwa.edu.au (P.B.); 2 Alzheimer’s Research Australia, Nedlands, Perth, WA 6009, Australia; 3 Faculty of Medicine, Human and Health Sciences, Macquarie University, Sydney, NSW 2109, Australia; 4 Faculty of Health and Medical Sciences, The University of Western Australia, Crawley, Perth, WA 6009, Australia; 5 Department of Food Science and Nutrition, College of Food and Agriculture Science, King Saud University, Riyadh 11451, Saudi Arabia; moorthy@ksu.edu.sa (R.K.); malshuniaber@ksu.edu.sa (M.A.A.); 6 Queensland Brain Institute, University of Queensland, St Lucia, QLD 4072, Australia; zhaoyu.li@uq.edu.au; 7 School of Molecular and Life Sciences, Curtin University, Perth, WA 6102, Australia; r.coorey@curtin.edu.au (R.C.); s.johnson@curtin.edu.au (S.K.J.); 8 Department of Nutrition and Food Science, Western Sydney University, Penrith, NSW 2751, Australia; v.jayasena@westernsydney.edu.au

**Keywords:** *Sorghum bicolor*, polyphenols, Alzheimer’s disease, amyloid beta, signalling pathways

## Abstract

Background/Objective: Accumulation of amyloid-beta (Aβ) senile plaques in the human brain is a major hallmark of Alzheimer’s disease (AD), which manifests as progressive decline in memory and cognitive functions and currently lacks effective disease-modifying therapies. Emerging evidence demonstrates that polyphenol-rich plant foods are potential complementary therapies for AD. Methods: In this study, we investigated crude polyphenol extracts (CPEs) and purified polyphenol extracts (PPEs) from three sorghum genotypes for their ability to inhibit Aβ_42_-induced toxicity in MC-65 cells. Thioflavin T fluorescence, cell viability, mitochondrial function, oxidative stress assays, and Western blotting, along with RNA sequencing and computational analyses, were used to characterise both functional and transcriptomic responses of the cells to polyphenol treatments. Results: CPEs and PPEs inhibited Aβ_42_ aggregation by 67–76% and significantly reduced Aβ oligomer species. The extracts increased cell viability against Aβ-induced toxicity by more than 70%, decreased intracellular oxidative stress, and enhanced mitochondrial activity by over 80%. Transcriptomic profiling revealed differential modulation of genes associated with ferroptosis and MAPK/NF- κB signalling pathways, indicating regulation of inflammatory and oxidative-stress responses are mechanisms underlying the observed neuroprotection. Conclusions: This study demonstrates that polyphenol extracts from black and red sorghum genotypes exert strong multitarget neuroprotection against Aβ_42_ toxicity in MC-65 cells. These findings support further evaluation of sorghum-derived polyphenols as complementary therapeutic candidates for AD, with in vivo studies required to establish efficacy and translational potential.

## 1. Introduction

Alzheimer’s disease (AD) is a progressive neurodegenerative disorder and the leading cause of dementia worldwide, with no effective disease-modifying treatment currently available. Central to pathology of AD are the accumulation of amyloid beta 42 (Aβ_42_) senile plaques, tau hyperphosphorylation, oxidative stress, mitochondrial dysfunction, and neuroinflammation. The multifactorial nature of AD has stimulated growing interests in multitarget therapeutic strategies, particularly those derived from natural products with minimal or no adverse effects [1].

Polyphenol-rich plant foods contain bioactive compounds with drug-like properties that modulate several pathological processes relevant to AD, including Aβ aggregation, oxidative damage, and neuroinflammation. Extracts from olive leaf [2], cinnamon [3], *Isodon japonicus* [4], *Allium roseum* [5], and various berries [6] have demonstrated potent inhibition of Aβ_42_ aggregation in vitro. Despite these promising findings, the therapeutic potential of *Sorghum bicolor*, a nutrient and polyphenol-rich cereal grain widely consumed in Asia and Africa, remains underexplored. Our research group was the first to comprehensively investigate the neuroprotective properties of *Sorghum bicolor* polyphenol extracts, demonstrating that extracts from six genotypes inhibit Aβ_42_ aggregation, tau phosphorylation, and Aβ-induced toxicity in BE-M17 neuroblastoma cells [7,8]. However, the molecular mechanisms underlying these protective effects have not been fully elucidated.

In the present study, we selected three *Sorghum bicolor* genotypes with the most potent neuroprotective properties from our earlier work [7,8]. From each genotype, we prepared both crude polyphenol extracts (CPEs) and purified polyphenol extracts (PPEs) and evaluated their effects on AD-related pathological processes, including Aβ_42_ aggregation and toxicity, oxidative stress, and mitochondrial dysfunction, using in MC-65 cells. By integrating biochemical assays with transcriptomic profiling, this work provides novel mechanistic insights into the neuroprotective potential of sorghum polyphenol extracts as complementary therapeutic candidates for AD.

## 2. Materials and Methods

### 2.1. Reagents

MC-65 cells, which are human central nervous system cell line that produce the C-99 fragment of the amyloid precursor protein (APP) under control of tetracycline-sensitive promoter, were kindly donated by Bryce Sopher of the University of Washington, USA. Transparent, black and white culture plates were purchased from Greiner Bio-One GmbH, Germany. Amyloid beta 42 (Aβ_42_) was obtained from ERI Amyloid laboratory, LLC (Oxford, CT, USA). Dulbecco’s modified eagle medium/F-12 (DMEM/F-12), GlutaMAX^TM^ supplement, Tetracycline (Tet), DMEM/F12 (no phenol red), Hank’s balanced salt solution (HBSS), Foetal Bovine serum (FBS), Geniticin Selective Antibiotic (G418) and Opti-MEM^TM^ were purchased from Thermo Fisher Scientific (New York, NY, USA). Dimethyl sulfoxide (DMSO), Trypsin (sequencing grade), and 1,1,1,3,3,3-Hexafluoro-2-propanol (HFIP) were purchased from Sigma Aldrich (St. Louis, MO, USA). Mitochondrial ToxGlo^TM^ was sourced from Promega (Alexandria, NSW, Australia), while Mitotracker (M22426), catalase (A22180) and peroxidase (A22188) were purchased from Molecular Probes^TM^ (Eugene, OR, USA). Ethanol and ethyl acetate were purchased from ChemSupply Australia (Gillman, SA, Australia). All other reagents used in this study were analytical grade.

### 2.2. Sample Preparation

Shawaya Short Black 1 (black genotype grain) was kindly donated by the Department of Agriculture and Fisheries, Brisbane, QLD, Australia. The red-brown genotype grain was purchased from a farm in Frankland, Western Australia. The powdered red genotype grain was purchased from a local market in Perth, Western Australia. The sorghum grains were separately ground into a fine powder using a NutriBullet Blender Combo 1200 (NutriBullet LLC, Shanghai, China). The powdered samples were then sealed in vacuum bags and stored at –20 °C in the dark until analysis.

#### 2.2.1. Crude Extraction of Polyphenols

Crude polyphenols extract was prepared as previously described [9]. Briefly, 1 g of each sorghum flour was separately mixed with 10 mL of 60% food-grade ethanol in a 250 mL volumetric flask. The mixture was incubated in a shaking water bath at 60 °C for 3 h. The supernatant was collected after centrifugation at 3220× *g* for 10 min. The residue was resuspended in 10 mL of fresh 60% food-grade ethanol, and the extraction process repeated twice. The supernatants were pooled and evaporated using a Buch rotary evaporator (BUCHI corporation, New Castle, DE, USA). Three crude polyphenol extracts (CPEs): crude black sorghum (CBS), crude red-brown sorghum (CRB), and crude red sorghum (CRS) were obtained. The slurry was resuspended in water, frozen, freeze-dried and stored at −30°C until analysis.

#### 2.2.2. Purification of Crude Polyphenol Extracts

Purified polyphenol extracts (PPEs) were obtained from the CPEs as previously described [10]. In brief, an aliquot of CPE from each sorghum genotype was mixed with ethyl acetate (1:4, *v*/*v*) and left to settle, allowing the top layer containing polyphenols to separate from the bottom layer containing waxes, terpenes, lipids, and other contaminants. The top layer was carefully pipetted into Falcon tubes, and the ethyl acetate purification was repeated three times. The top layers were pooled and concentrated by evaporating the ethyl acetate using a Buchi rotary evaporator, yielding three purified polyphenol extracts (PPEs): purified black sorghum (PBS), purified red-brown sorghum (PRB), and purified red sorghum (PRS). The slurry was resuspended in water, frozen, freeze-dried and stored at −30 °C until analysis.

### 2.3. Peptide Preparation

Amyloid beta peptide (Aβ_42_) was prepared as previously described [11]. Briefly, the dry peptide was weighed to 1.8 mg and dissolved in 500 µL of 1,1,1,3,3,3-hexafluoro-2-propanol (HFIP) at room temperature (RT) for 1 h. The resulting solution was aliquoted into 450 µg portions and allowed to dry overnight at RT. The dried peptide films were stored at −20 °C until analysis. For the Thioflavin T aggregation assay, the peptide films were dissolved in DMSO and sonicated at 18 °C for 15 min (Bioruptor^®^Plus, Diagenode Incorporated, Denville, NJ, USA). The peptide solution was adjusted to 100 µM by the addition of 980 µL ice cold Ham’s F-12 phenol red-free media.

### 2.4. Thioflavin T Aggregation Assay

Aβ_42_ peptides were prepared as described above, and aggregation kinetics were assessed at a final concentration of 20 µM [12]. Different concentrations of each polyphenol extract (CBS, CRB, CRS, PBS, PRB, and PRS) were prepared at 50, 100, 150, 250, and 500 µg/mL in Tris-buffered saline (pH 7.4). Quercetin, used as a positive control, was prepared at concentrations of 20–50 µg/mL in the same buffer. For the assay, 80 µL of each extract was added to individual well of a black, clear bottom 96-well plate, followed by 30 µL of the prepared Aβ_42_ peptide solution and 8 µL of 6 µM ThT solution. A negative control consisted of 30 µL of DMSO-F12 solution in place of Aβ_42_. Plates were incubated in a PerkinElmer multimode plate reader (PerkinElmer, Waltham, MA, USA) for 23 h, and fluorescence was measured with excitation and emission maxima set to 450 nm and 490 nm, respectively. The assay was performed in triplicate, with each condition tested in five technical replicates for both CPEs and PPEs in combination with Aβ_42_.

### 2.5. Cell Viability Assay

The cell viability assay was performed as previously described [13,14]. MC-65 cells were cultured in DMEM supplemented with heat-inactivated FBS, 5 µg/mL tetracycline and 4 mg/mL G418 and maintained at 37 °C in a humidified incubator with 5% CO_2_. Stock solutions of each polyphenol extract (20 mg/mL) were prepared in DMSO. For the assay, 100 µL of MC-65 cells (5 × 10^4^ cells/well) were seeded into 96-well plates in fresh complete medium and incubated for 72 h. The medium was then replaced with 100 µL Opti-MEM containing either crude polyphenol extracts (CPEs: 250–750 µg/mL), purified polyphenol extracts (PPEs: 250–750 µg/mL), or quercetin (125–250 µg/mL). Tetracycline (4 µg/mL) served as the positive control. After a further 72 h incubation, 20 µL of MTS solution (3-(4,5-dimethylthiazol-2-yl)-5-(3-carboxymethoxyphenyl)-2-(4-sulfophenyl)-2H-tetrazolium) was added to each well, followed by a 4 h incubation under the same conditions. Absorbance was measured at 570 nm using a PerkinElmer multimode plate reader (PerkinElmer, Waltham, MA, USA), and cell viability was expressed as a percentage of the tetracycline control.

### 2.6. Determination of Intracellular Adenosine Triphosphate Levels

The three most potent extracts identified in the toxicity assay (250 µg/mL CBS, PBS, and PRS) were selected for mitochondrial function analysis. Mitochondrial ATP levels were measured using the ToxGlo™ Mitochondrial Toxicity Assay Kit following the standard protocol. Briefly, confluent MC-65 cells were harvested and seeded at 5 × 10^4^ cells/well in complete medium in Nunc^TM^ clear bottom, white opaque 96-well plates, with or without tetracycline, and incubated for 72 h at 37 °C in 5% CO_2_. The medium was then replaced with Opti-MEM^TM^ containing quercetin (125 µg/mL), polyphenol extracts (250 µg/mL CBS, PBS, or PRS), tetracycline, mitochondrial toxins [digitonin (800 µg/mL) or sodium azide (NaN_3_, 50 µM)]. After 90 min incubation at 37 °C, 20 µL of 5× Cytotoxicity Reagent was added to each well and incubated for 30 min. Fluorescence was recorded at Ex/Em 485/525 nm using a VANTAstar plate reader (BMG LABTECH, Ortenberg, Germany). Subsequently, 100 µL of ATP Detection Reagent was added to each well at RT, shaken for 5 min, and luminescence was recorded on the same instrument. The assay was also repeated with a 72 h treatment period prior to ATP measurement. ATP levels were expressed as a percentage relative to no-tetracycline controls.

### 2.7. Live Cell Microscopy

MC-65 cells were seeded in 12-well plates at 5 × 10^5^ cells/well as described above. After 72 h, polyphenol-treated, tetracycline (Tet), and no-tetracycline (No Tet) control cells were incubated with 150 nM MitoTracker™ Deep Red at 37 °C in 5% CO_2_ for 30 min. The stain was removed, and cells were washed three times with phosphate-buffered saline (PBS; pH 7.4) before fluorescence measurement (Ex/Em 644/665 nm) using a VANTAstar plate reader (BMG LABTECH, Ortenberg, Germany), followed by imaging with a Nikon Eclipse Ti2-E wide-field fluorescence microscope (Nikon, Tokyo, Japan).

### 2.8. Antioxidant Enzymes Activities

Cells were cultured and treated with or without polyphenol extracts for 72 h as described above. Catalase and peroxidase activities were measured using the Amplex^®^ Red Catalase Assay Kit (A22180) and Amplex^®^ Red Hydrogen Peroxide/Peroxidase Assay Kit (A22188) following the standard protocol. Luminescence was recorded using a VANTAstar plate reader (BMG LABTECH, Ortenberg, Germany).

### 2.9. Detection of Intracellular Oxidative Stress

Intracellular reactive oxygen species (ROS) were measured using the CellRox^®^ Orange reagent (C10422). MC-65 cells were seeded in 6-well plates at a density of 1 × 10^6^ cells/well in DMEM until confluent. The medium was then replaced with Opti-MEM containing tetracycline, 250 µg/mL of each extract, or 125 µg/mL quercetin, and cells were incubated for 72 h at 37 °C in a humidified atmosphere with 5% CO_2_. After incubation, the cells were washed with PBS and treated with fresh medium containing 5 µM CellRox^®^ Orange for 30 min under the same conditions. The stain was removed, and the cells were washed three times with PBS. Brightfield and fluorescence images were captured at 20× magnification using a Nikon Eclipse Ti2-E wide-field fluorescence microscope (Nikon, Tokyo, Japan).

### 2.10. Estimation of Aβ Clearance by Western Blotting

Clearance of Aβ species from MC-65 cells was assessed as previously described [15]. Briefly, confluent cells in DMEM were resuspended in Opti-MEM and seeded in 6-well plates at a density of 1 × 10^6^ cells/well. Treatments were Opti-MEM with or without tetracycline (Tet), 250 µg/mL CBS, PBS, or PRS extracts, or 125 µg/mL quercetin. Plates were incubated for 72 h at 37 °C in a humidified atmosphere containing 5% CO_2_. Following incubation, cells were harvested, washed with HBSS, and lysed in Tricine buffer. Lysates were centrifuged at 12,000× *g* for 5 min, and protein concentrations in the supernatant were determined using the Bradford assay [16]. Exactly 12 µg of protein from each treatment was loaded onto NuPAGE™ Tris gels (Thermo Fisher Scientific, USA) alongside 12 µL of Novel Sharp Pre-stained Protein Standard. Electrophoresis was performed in 1× MES Running Buffer at 110 V for 1 h, followed by transfer onto nitrocellulose membranes using the Trans-Blot Turbo Transfer System (Bio-Rad Laboratories, CA, USA). Membranes were blocked with 5% (*w*/*v*) milk in Tris-buffered saline (TBS) for 1 h at RT. For Aβ detection, membranes were incubated overnight at 4 °C with 5 µL anti-Aβ_42_ (6E10) antibody in 10 mL of 0.5% TBS-T. For loading-control detection, membranes were incubated overnight with GAPDH antibody. After three washes in 1× TBS-T (8 min each), membranes were incubated for 1 h in 10 mL TBS-T containing 2 µL of secondary anti-mouse antibody (for Aβ_42_) or anti-rabbit antibody (for GAPDH). Final washes were performed twice in TBS-T (8 min each), then twice in TBS (8 min each). About 1 mL of an equal-volume mixture of Amersham™ ECL™ Western blotting Reagents 1 and 2 (GE Healthcare Bio-Sciences, Uppsala, Sweden) was introduced on the membrane before imaging using a VILBER Fusion FX Spectra 6.0 EDGE chemiluminescence system (Vilber, Collégien, France). Band intensities were quantified using Image Lab software, version 6.1.0 (Bio-Rad Laboratories, Hercules, CA, USA).

### 2.11. Next Generation Standard RNA Sequencing

After reaching confluence in DMEM, the cells were suspended in Opti-MEM and seeded in 6-well plates (1 × 10^6^ cells/well) with or without Tet., 250 µg/mL CBS, PBS, PRS or 125 µg/mL quercetin. The plate was incubated in a humidified atmosphere containing 5% CO_2_ at 37 °C for 72 h. Purified total RNA was extracted from the cells using ReliaPrep miniprep systems (Promega, Madison, WI, USA) following standard protocol. The extracted RNA was quantified using Denovix DS-11 Series spectrophotometer (Denovix, Wilmington, DE, USA). mRNA library construction, sequencing and differential gene-level expression analyses were performed as previously described [17,18].

### 2.12. Validation of Identified Genes Using Bioinformatics Computation

#### 2.12.1. Data Mining

The dataset related to AD (GSE5281) was sourced from the Gene Expression Omnibus (GEO) database using the relevant query criteria. The gene expression profile was retrieved and downloaded from the dataset. GSE5281 contains hundreds of normal and AD brain samples to study differentially expressed genes at the transcriptional level.

#### 2.12.2. Identification of Critical Targets from AD Database

The GEO2R resource within GEO was used to identify potent targets within the GSE5281 dataset. The GEO2R resource offers criteria and visualisation tools which enhanced accurate identification of significant genes and pathways through their expression profiles. This enabled identification of genes relevant to AD pathology, as well as potential molecular mechanisms underlying AD pathology.

#### 2.12.3. Drug–Protein Interaction Network

For a streamlined bioinformatics study, quercetin, a flavonoid identified in the *Sorghum bicolor* polyphenol extracts (CBS, PBS and PRS), was selected as the representative compound for this analysis. Overlapping genes associated with both quercetin and AD were retrieved from the Comparative Toxicogenomics Database (CTD) and GeneCards. Data from these overlapping genes were used to identify potential targets involved in AD and evaluate efficacy of quercetin. Binding affinities and interactions between quercetin and the target genes were predicted using computational techniques. Target genes were entered into search tools for interacting chemicals (STITCH) within Cytoscape 3.10.4 platform, to construct quercetin–protein interaction network.

#### 2.12.4. Gene Ontology and Pathway Enrichment Analysis

The *g:profiler* database (https://biit.cs.ut.ee/gprofiler/home, accessed on 15 March 2025) was queried to deepen understanding of the biological functions of the potent genes and enriched pathways. In addition, Reactome and Kyoto Encyclopaedia of Genes and Genomes (KEGG) pathway analyses (https://www.kegg.jp/kegg/, accessed on 10 April 2025) were performed to identify specific pathways presenting strong enrichment with the identified target genes. These analyses identified genes involved in critical cellular functions and their links with AD-related pathways.

#### 2.12.5. Protein Preparation

Three-dimensional structures of the target proteins were retrieved from the Protein Data Bank (PDB). Minor regularisation was performed to reconstruct missing side chains using the “Construct/Check/Repair Structure” and “Prepare the Database File for Docking Programs” modules. In addition to correcting the positions of water molecules and ensuring proper symmetrical alignment, hydrogen atoms were added. For preparation of the Protein Data Bank with Partial Charges and AutoDock Atom Types (PDBQT) files, AutoDock Tools (ADT, version 1.5.4) was used. Gasteiger charges for protein atoms were then calculated using ADT.

#### 2.12.6. Ligand Training

The antioxidant vitamins C and E (ascorbic acid and α-tocopherol) were used as reference ligands. Their two dimensional (2D) chemical structures were constructed using the software programme ChemSketch 12.01. Geometry optimisation was performed to reduce energy and generate very stable three-dimensional (3D) conformations of each compound. This optimisation enhanced the precision of bond angles, bond lengths, and torsional conformations before downstream computational modelling. Partial atomic charges were assigned to each ligand using the default force-field parameters in the ChemSketch 12.01 programme. The optimised structures were saved as mol2 files to preserve the 3D coordinates, assigned charges, and atom types. Subsequently, the mol2 files were imported into ADT for further ligand preparation steps.

#### 2.12.7. Docking Method

A total of 100 runs of the Lamarckian Genetic Algorithm with local search were performed as the docking search strategy. Cluster analysis was subsequently applied to the docking results. The interactions between quercetin (CID: C012526) and the selected target proteins identified from the RNA sequencing analysis were evaluated, including c-Jun N-terminal kinase (JUN) (PDB: [ID]), Nuclear Factor Kappa-Light-Chain-Enhancer of Activated B cells (NF-κB) (PDB: [ID]), receptor tyrosine kinase-like orphan receptor 2 (ROR2) (PDB: [ID]), cyclooxygenase-2 (COX-2) (PDB: [ID]), complement component 5a receptor 1 (C5AR1) (PDB: [ID]), protein phosphatase 1 regulatory subunit 15A (PPP1R15A) (PDB: [ID]), and ferroptosis suppressor protein 1 (FSP1) (PDB: [ID]). Protein structures were retrieved from the PDB. Docking results were visualised and analysed using BIOVIA Discovery Studio, enabling molecular-level interpretation of binding interactions.

### 2.13. Statistical Analysis

All data were presented as means ± SEM of three independent experiments. Data were analysed by one-way ANOVA using GraphPad Prism software version 10.4.1 (GraphPad Software, San Diego, CA, USA). Tukey’s post hoc multiple-comparison test was applied to identify significant differences among treatment means. Significant differences were considered at *p* ≤ 0.05. For molecular docking and bioinformatics, Advanced Chemistry Development, Inc. ChemSketch 12.01 was used to prepare the ligands. HyperChem 8.0.3 was utilised to optimise the geometrical representations, and the Wizard of AutoDock Tools 1.5.4 was used to prepare the proteins for this work.

## 3. Results

### 3.1. Thioflavin T Fluorescence

**Figure 1:**
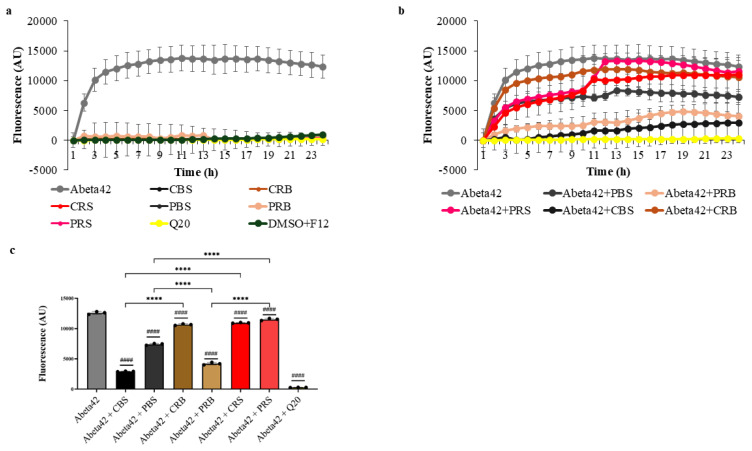
Inhibitory effects of sorghum polyphenol extracts on amyloid beta 42 (Aβ_42_) aggregation. (**a**) Self aggregation kinetics of Aβ_42_, 50 µg/mL CBS, CRS, CRB, PBS, PRB, PRS, or 20 µg/mL quercetin along with DMSO+F-12 control, assessed by Thioflavin T fluorescence assay over a 23 h period. (**b**) Aggregation kinetics of 20 µM Aβ_42_ co-incubated with polyphenol CBS, CRS, CRB, PBS, PRB, PRS or 20 µg/mL quercetin over 23 h period. Thioflavin T fluorescence is presented as arbitrary units (AU). (**c**) Total fluorescence quantification at 23 h. All the polyphenol extracts produced significantly lower fluorescence than Aβ_42_. Data are presented as mean ± SD (*n* = 3). Statistical significance: **** *p* < 0.0001 (between treatments); ^####^ *p* < 0.0001 (compared with Aβ_42_). CBS: crude black sorghum; CRB: crude red-brown; CRS: crude red sorghum; PBS: purified black sorghum; PRB: purified red-brown; PRS: purified red sorghum, Q20: quercetin.

The effects of sorghum crude polyphenol extracts (CPEs) and purified polyphenol extracts (PPEs) on Aβ_42_ aggregation kinetics were assessed using the Thioflavin T (ThT) fluorescence assay. ThT, a benzothiazole dye that exhibits enhanced fluorescence upon binding to Aβ_42_ aggregates, is widely used to monitor amyloid fibril formation in vitro in the presence of anti-amyloidogenic compounds [3]. Initial assays were performed separately with Aβ_42_, CPEs, PPEs, or quercetin (20 µg/mL; positive control) (Figure 1a). Fluorescence values measured over 0–23 h were normalised by subtracting baseline readings at t = 0, allowing calculation of the relative increase in fluorescence over time. Aβ_42_ alone produced a sigmoidal aggregation curve at 37 °C [19], with a sharp increase in fluorescence between 0 and 4 h, a slower rise peaking at 11 h, and a subsequent plateau (final fluorescence = 12,346.2 AU) (Figure 1a).

In contrast, all polyphenol extracts showed significantly suppressed self-aggregation (Figure 1a), characterised by a slight increase in fluorescence and a delayed peak occurring between 13 and 23 h (final fluorescence = 213.6–964 AU). To further examine their inhibitory potential, Aβ_42_ was separately co-incubated with serial dilutions (50–500 µg/mL) of CPEs or PPEs, and with quercetin (20 µg/mL), a well-established inhibitor of Aβ_42_ aggregation [20,21]. Data from extract concentrations of 100–500 µg/mL were excluded from quantitative analysis due to colour interference that generated negative fluorescence values, likely caused by spectral overlap or quenching by extract pigments, a common limitation of fluorescence-based assays [22]. As shown in Figure 1b, Aβ_42_ alone exhibited a typical sigmoidal aggregation profile, with fluorescence rising rapidly between 0 and 4 h, peaking at ~15,000 AU by 11 h, and stabilising thereafter. In contrast, all the sorghum CPEs and PPEs significantly delayed and reduced fibril formation, shifting aggregation peaks to 13–23 h, and lowering final to 213.6–964 AU. PBS and CBS demonstrated the strongest inhibition, maintaining consistently low fluorescence over the time course, followed by PRB. CRS and PRS were less effective. Quercetin completely suppressed Aβ_42_ aggregation, maintaining baseline fluorescence. At 50 µg/mL, all extracts except PRS, reduced fluorescence by 67.15–76.18% (*p* < 0.0001), whereas PRS showed only 8.4% reduction. End point measurements at 23 h confirmed significantly lower fluorescence for all extracts compared with Aβ_42_ alone. The overall Aβ_42_ aggregation inhibition ranking was CPEs < PPEs < quercetin, with black sorghum extracts displaying the most pronounced anti-amyloid activity (Figure 1c). These findings demonstrate that sorghum polyphenols extracts, particularly those from black genotypes, exert strong inhibitory effects on Aβ_42_ fibril formation in vitro.

### 3.2. CPEs and PPEs Inhibit Aβ-Induced Toxicity in MC-65 Cells

**Figure 2:**
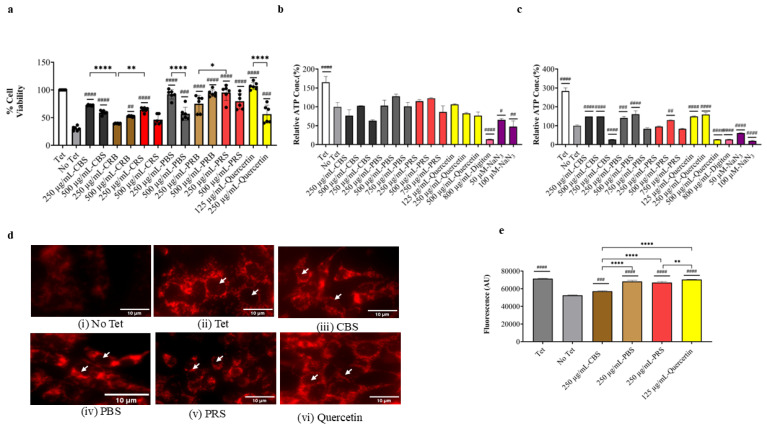
Neuroprotective effects of sorghum polyphenol extracts on amyloid beta (Aβ)-induced neurotoxicity. (**a**) Effect of CBS, CRS, CRB, PBS, PRB, PRS, and quercetin on Aβ42-induced toxicity in APP-C99-expressing MC-65 cells cultured in Opti-MEM for 72 h with 250–500 µg/mL extract. Cells grown without or with tetracycline served as negative and positive controls, respectively. Viability was quantified by MTS. All extracts significantly attenuated Aβ-induced toxicity in a dose- and genotype-dependent manner. (**b**,**c**) Relative ATP abundance following treatment with three different doses of CBS, PBS, PRS or quercetin, measured by Mitochondrial ToxGlo™ assay at (**b**) 1.5 h and (**c**) 72 h. All treatments differentially increased ATP levels by between 49.5 and 61.09%, compared with No Tet. (**d**,**e**) Mitochondrial network integrity and mass visualised with Mitotracker^®^ Deep Red after treatment with 250 µg/mL polyphenols or 125 µg/mL quercetin. Cells were stained with 150 nM Mitrotracker dye, incubated for 30 min at 37 °C, and washed three times with pre-warmed culture medium. (**d**) Representative live images (20× objective); white arrows indicate cell bodies. (**e**) Quantification of total mitochondrial fluorescence by plate reader; fluorescence intensity indicates level of mitochondrial activity. No Tet. cells show clustered, fragmented mitochondrial with reduced fluorescence. Data are mean ± SEM (*n* = 3). Significance: * *p* < 0.05, ** *p* < 0.01, **** *p* < 0.0001 (between treatments); ^#^ *p* < 0.5, ^##^ *p* < 0.01, ^###^ *p* < 0.001, ^####^ *p* < 0.0001 (compared with No Tet). Tet: Tetracycline; CBS: crude black sorghum; CRB: crude red-brown; CRS: crude red sorghum; PBS: purified black sorghum; PRB: purified red-brown; PRS: purified red sorghum; ATP: adenosine triphosphate.

The MTS assay was used to determine whether the extracts could attenuate Aβ-induced toxicity in MC-65 cells. The cells were treated with or without 250 or 500 µg/mL of CPEs or PPEs, or with 125–250 µg/mL quercetin as a control in reference to our published studies [7,8]. All PPEs strongly inhibited Aβ-induced toxicity compared with CPEs (*p* < 0.0001). Cells survival ranged from 42 to 77% for CPEs and 64–100% for PPEs (Figure 2a).

Among the CPEs, CBS produced the strongest neuroprotection at both concentrations (59–72% viability), followed by CRS (46–64%), and CRB (39–52%). All PPEs strongly inhibited Aβ-induced neurotoxicity, particularly at 250 µg/mL. At both concentrations, PBS, PRB and PRS treatments resulted in 56–92%, 75–94%, and 79–95% cell viability, respectively (Figure 2a). Quercetin (125–250 µg/mL) increased cell viability by 56–107% (*p* < 0.0001) (Figure 2a). Higher concentrations of CPEs or PPEs (750 and 1000 µg/mL) did not show any pharmacological improvement compared with the No Tet. cells (Table S1). Collectively, these findings indicate that the polyphenol extracts inhibited Aβ-induced neurotoxicity in a concentration- and genotype- dependent manner. Based on these results, the lowest concentrations that significantly enhanced cell viability (250 µg/mL for CBS, PBS, PRS, and 125 µg/mL for quercetin) were selected for subsequent experiments.

### 3.3. CPEs and PPEs Restored ATP Levels in MC-65 Cells

The ATP assay was employed to further measure Aβ-mediated toxicity and to evaluate the protective effects of sorghum polyphenol extracts in MC-65 cells. ATP levels at 1.5 h and 72 h were expressed as a percentage relative to No Tet. cells following the standard protocol (Mitochondrial ToxGlo, Promega, Madison, WI, USA). In No Tet. controls, ATP showed a slight decrease at 1.5 h, which progressed to a substantial decline after 72 h, consistent with intracellular Aβ accumulation and impaired mitochondrial activity (Figure 2b,c). Treatment with a single or double doses of polyphenol extracts (250 µg/mL or 500 µg/mL) inhibited ATP decline in a dose- and genotype-dependent manner. At 1.5 h, PBS and PRS increased ATP levels by up to 32.95% and 22.70%, respectively (Figure 2b); whereas, CBS at 500 µg/mL showed a slight increase (2.31%). The limited ATP recovery observed with CBS may be due to the presence of cytotoxic components or impurities in crude extracts, including pigments, polysaccharides, chlorophylls, waxes, oxidised polyphenols, or quinones [23,24,25]. As expected, mitochondrial toxins digitonin (800 µg/mL) and sodium azide (100 µM) caused marked ATP depletion of 86.2% and 80.23%, respectively (*p* < 0.0001). At 72 h, intracellular Aβ accumulation significantly reduced ATP levels in No Tet. cells compared with all treatment groups (*p* < 0.0001) (Figure 2c). Polyphenol extracts restored ATP in a manner dependent on concentration, sorghum genotype, and exposure duration. PBS at 250 µg/mL (61.09%) and quercetin (59.27%) at 125 µg/mL were the most effective, followed by CBS (49.54%) and PRS (14.29%). In contrast, higher doses (750 µg/mL) of all extracts were cytotoxic, reducing ATP levels by 17–72%. Digitonin and sodium azide decreased ATP levels by 72.63% and 52.59%, respectively. These findings showed that sorghum polyphenol extracts were neuroprotective against Aβ-induced mitochondrial dysfunction. Based on this, we next examined how the treatments influenced mitochondrial activity and morphology as potential mechanisms underlying ATP restoration.

### 3.4. CPEs and PPEs Increased Mitochondrial Activity by Colocalisation in MC-65 Cells

Live cell mitochondria were visualised using the mitochondria-specific fluorescent MitoTracker^®^ Deep Red dye to assess network integrity and activity. Fluorescence intensity corresponds to abundance of active mitochondria. In No Tet. cells, intracellular Aβ_42_ disrupted the reticular mitochondrial network, producing small, rounded specks indicative of fragmentation (Figure 2(di)). By contrast, Tet. cells displayed abundant mitochondrial mass with ridge-like morphology and an interconnected tubular network (Figure 2(dii)). Treatment with CBS, PBS, PRS, or quercetin preserved the reticular mitochondrial network in the cell body (Figure 2(diii–vi)). Quantitative analysis showed a significant reduction in total MitoTracker fluorescence per cell in No Tet. cells compared with Tet. and all treated groups (98.54–108.69% of Tet.; *p* < 0.0001) (Figure 2e). Among the treatments, PBS produced the strongest mitochondrial activity, followed by PRS and CBS, with quercetin achieving a fluorescence comparable to PBS.

### 3.5. Effects of CEs and PPEs on Intracellular ROS Production

**Figure 3:**
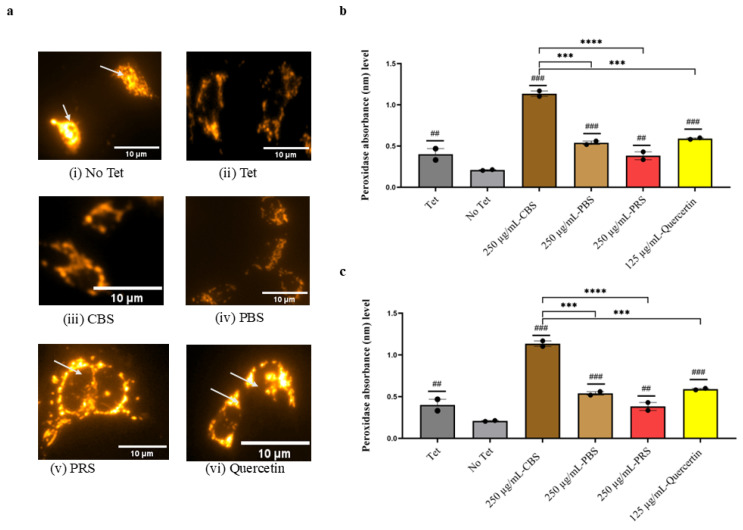
Sorghum polyphenol extracts treated attenuate oxidative stress and restore antioxidant enzyme activity. (**a**) MC-65 cells were treated with or without tetracycline, 250 µg/mL polyphenols or 125 µg/mL quercetin for 72 h, then labelled with CellRox Orange and imaged. Intense orange fluorescence indicates localisation of ROS in the nuclear and in the perinuclear membrane (**a**) i–vi. White arrows indicate cells under oxidative stress. (**b**,**c**) Antioxidant enzyme activities following 72 h treatment with or without polyphenol extracts, quantified using Amplex Red Catalase and Peroxidase Assay kit. (**b**) Catalase activity; (**c**) peroxidase enzyme activity. The CBS and PBS elicited significant increases in the enzyme activities. Results are presented as mean ± SD. Significance: *** *p* < 0.001, **** *p* < 0.0001 (between treatments); ^##^ *p* < 0.01, ^###^ *p* < 0.001, (compared with No Tet). Tet: Tetracycline; CBS: crude black sorghum; PBS: purified black sorghum; PRS: purified red sorghum.

Elevated oxidative stress, driven by reactive oxygen species (ROS), is implicated in AD pathogenesis, occurring before and during accumulation of Aβ_42_ [26]. The ROS-sensitive dye CellROx^®^ Orange was used to measure oxidative stress in live MC-65 cells. CellROx^®^ Orange is non-fluorescent in its reduced state but emits bright orange fluorescence when oxidised by ROS, after which it localises to the nucleus and mitochondria through DNA binding. Figure 3 shows representative images of ROS generation across all treated and No Tet. cells. In the presence of Aβ, No Tet. cells produced intense signal in the nuclear regions, indicative of elevated ROS accumulation (Figure 3(ai)). However, all treated cells showed low fluorescence intensities, implying reduced intracellular ROS levels (Figure 3(aii–avi)).

### 3.6. CEs and PPEs Enhanced Levels of Catalase and Peroxidase Enzymes

Endogenous antioxidant defences are depleted in AD, with decreased activities of catalase (CAT), peroxidase (PER), and superoxide dismutase, thereby impairing the neurons ability to neutralise ROS [27]. Aβ-producing No Tet. cells exhibited marked depletion of CAT (136.3 mU/mL) and PER (0.209 nm) compared with Tet. controls (Figure 3b,c). Treatment with tetracycline, CBS, PBS, PRS, or quercetin for 72 h significantly enhanced CAT and PER activities in a genotype-dependent manner. CAT activity was most strongly enhanced by CBS (60.5%), followed by PBS (26.3%), quercetin (24.5%), and PRS (13.9%) (Figure 3b). Similarly, all treatments, except PRS, caused around two-fold increase in PER activity.

### 3.7. CEs and PPEs Promote Clearance of Intracellular Amyloid Beta (Aβ)

**Figure 4:**
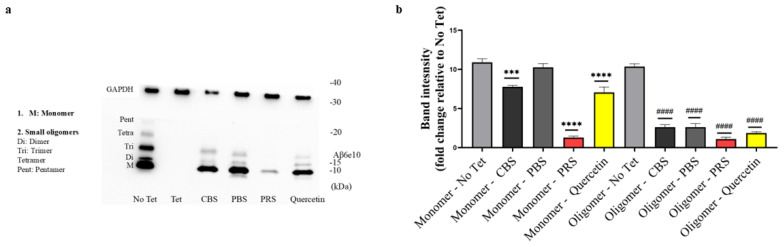
Polyphenol extracts disrupted Aβ_42_ aggregation in MC-65 cells. The cells were seeded in 6-well plates (Opt-MEM medium; 1 × 10^6^/well) and cultured for 72 h in the presence or absence of tetracycline or polyphenol extracts. Cell lysates were prepared, and 20 µg protein per lane were resolved by SDS-PAGE and analysed by Western blot. (**a**) Representative immunoblot of Aβ_42_ species under Tet. withdrawal (No Tet.) and in the presence of Tet., CBS, PBS, PRS or quercetin. (**b**) Quantification of Aβ_42_ monomer and oligomer. No Tet. cells showed more Aβ_42_ dimers, oligomer, trimers and tetramers, which were significantly reduced in Tet-maintained and polyphenols-treated cells. Data are mean ± SD. Significance: *** *p* < 0.001, **** *p* < 0.0001 (compared with No Tet. Monomer); ^####^ *p* < 0.0001 (compared with No Tet. oligomer); Tet: Tetracycline; CBS: crude black sorghum; PBS: purified black sorghum; PRS: purified red sorghum.

Excessive generation of Aβ and the accumulation of soluble Aβ_42_ oligomers are widely recognised as critical drivers of AD onset and progression [28]. In the presence of tetracycline, Aβ_42_ species was undetectable; tetracycline withdrawal caused a significant increase in higher-molecular-weight small oligomers (dimers, trimers, and tetramers) in No Tet. cells (*p* < 0.0001) (Figure 4a,b). Treatments with CBS, PBS, PRS, or quercetin significantly reduced the abundance of these oligomers by 75%, 77%, 89%, and 83%, respectively (*p* < 0.0001), while monomer levels remain largely unchanged. CBS and PBS achieved reductions comparable to quercetin, whereas PRS showed the most oligomer clearance effect. The accumulation of Aβ oligomers in No Tet. cells correlated with the observed decline in cell viability after three days (Figure 2a), suggesting that the neuroprotective effects of sorghum polyphenols are mediated, at least partly, through inhibition of toxic Aβ_42_ oligomer formation.

### 3.8. Sorghum Polyphenol Extracts Modulate MAPK/NF-κB and Ferroptosis Pathway

**Figure 5:**
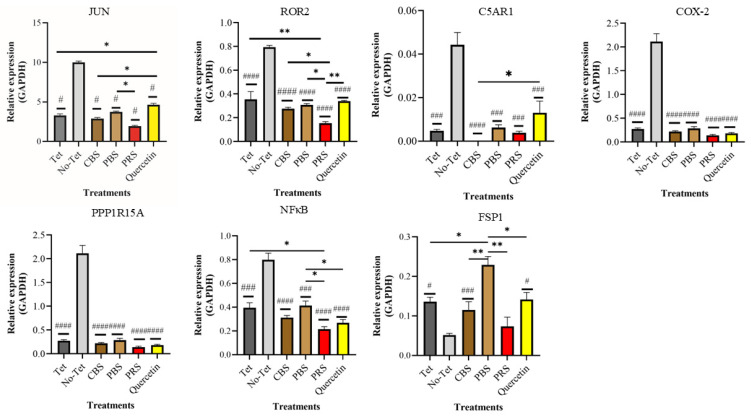
Sorghum polyphenol extracts differentially regulated expression of MAPK/NF-κB and ferroptosis-associated genes in MC-65 cells. Cells were cultured with or without Tet., and treated with 250 µg/mL CBS, PBS, PRS or 125 µg/mL quercetin, followed RNA-sequencing-based transcript quantification (gene profiling). Each transcript level was normalised to GAPDH. There was downregulation of genes (**a**) JUN (**b**) ROR2 (**c**) C5AR1 (**d**) COX-2 (**e**) PPP1R15A, and (**f**) NF-κB, and (**g**) upregulation of FSP1. JUN: c-Jun N-terminal kinase; ROR2: Receptor Tyrosine Kinase-Like Orphan Receptor 2; C5AR1: Complement Component 5a Receptor 1; COX- 2: Cyclooxygenase-2; PPP1R15A: Protein Phosphatase 1 Regulatory Subunit 15a; NF-κB: Nuclear Factor Kappa-Light-Chain-Enhancer of Activated B cells; FSP1: Ferroptosis Suppressor Protein 1. Data are mean ± SD. Statistics: * *p* < 0.05, ** *p* < 0.01, (between treatments); ^#^ *p* < 0.5, ^###^ *p* < 0.001, ^####^ *p* < 0.0001 (compared with No Tet). Tet: Tetracycline; CBS: crude black sorghum; PBS: purified black sorghum; PRS: purified red sorghum.

To explore mechanisms underlying the neuroprotection, we studied AD-associated transcriptional responses of polyphenol-treated cells. From the large dataset, we focused on Mitogen-activated protein kinase (MAPK), Nuclear Factor Kappa-Light-Chain-Enhancer of Activated B cells (NF-κB), and ferroptosis-related genes due to their roles in oxidative-stress-mediated neuronal damage [29] and iron-dependent cell death [30]. In No Tet. cells, MAPK/NF-κB dependent genes, including JUN, ROR2, C5AR1, COX-2, PPP1R15A, and NF-κB, were significantly upregulated compared with Tet. controls (Figure 5a–f); whereas, ferroptosis-suppressor (FSP1) was greatly downregulated. Treatment with tetracycline, 250 µg/mL CBS, PBS, PRS, or 125 µg/mL quercetin significantly downregulated the expression of all MAPK/NF-κB-associated genes, accompanied by significant upregulation of FSP1. CBS and PBS produced the most pronounced suppression of pro-inflammatory genes, while quercetin achieved the highest upregulation of FSP1. These gene modulation suggest sorghum polyphenol extracts attenuate Aβ_42_-induced inflammatory and oxidative-stress signalling while enhancing ferroptosis suppression, providing a mechanistic basis for their neuroprotective potential in MC-65 cells.

### 3.9. Interaction of Quercetin with MAPK/NF-κB/Ferroptosis Associated Genes

#### 3.9.1. Identification of the Target Genes and Pathways in AD

To further investigate the potential interaction of quercetin, a flavonoid present in CBS, PBS, and PRS, with MAPK/NF-κB/ferroptosis associated genes, AD related transcriptional profiles were analysed using the GSE5281 dataset from the GEO database. Comprehensive bioinformatics and statistical analyses were performed using GEO2R with the limma package to identify potent gene targets and pathways associated with AD. Volcano plots (Figure S1a,b) revealed numerous differentially expressed genes, with significant subsets upregulated (red) or downregulated (blue) in AD brains compared to normal controls, while others showed no significant change (black). Expression density analysis (Figure S1c) demonstrated a clear shift in transcript distribution between AD and control samples, consistent with widespread transcriptional dysregulation. Box plot analysis (Figure S1d) further confirmed strong variation in the expression levels of multiple target genes with distinct patterns between normal (green) and AD (purple) samples. These analyses identified several MAPK/NF-κB/ferroptosis-associated genes that were significantly altered in AD and are known or predicted targets of quercetin.

#### 3.9.2. Quercetin Interacts with AD Associated Genes

**Figure 6:**
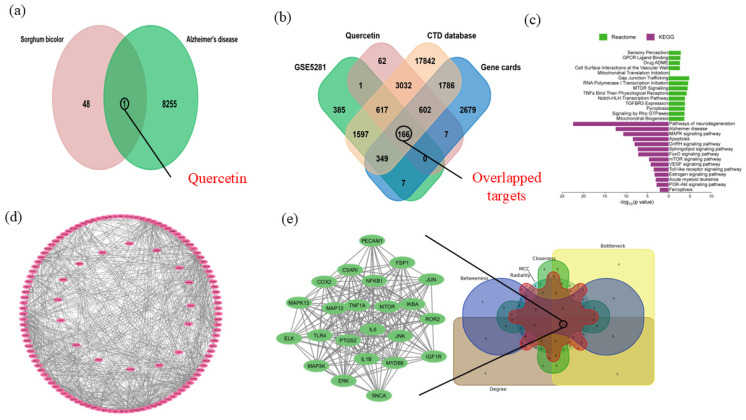
Protein–protein interaction (PPI) network and module discovery of quercetin-associated pathway against Alzheimer’s disease (AD). (**a**) Quercetin overlaps *Sorghum bicolor* metabolites with AD in database screening. (**b**) Multi-database target integration (GSE5281, CTD, and Gene cards). (**c**) Top 14 enriched pathways associated with AD (Reactome and KEGG). (**d**) STRING PPI network, curated in Cytoscape. (**e**) Fifteen potential targets identified by Cytohubba and MCODE.

Quercetin has been identified in several sorghum genotypes contributing to its health-promoting properties. Database screening identified quercetin as a polyphenol linking sorghum metabolites to potential AD intervention (Figure 6a).

To determine putative interaction, we integrated targets from GSE5281 dataset, the Comparative Toxicogenomics Database (CTD), and Gene Cards, yielding 166 overlapping AD-associated potential targets predicted to interact with quercetin (Figure 6b). Pathway enrichment (Reactome and KEGG) revealed 14 significantly AD-related pathways, including MAPK/NF-κB signalling, oxidative stress regulation, apoptosis, ferroptosis, and inflammatory responses (Figure 6c). A protein–protein interaction (PPI) network of these targets (STRING, visualised in Cytoscape) highlighted MAPK/NF-κB pathway signalling (Figure 6d). Hub-gene analysis (CytoHubba) and module detection (MCODE) identified 15 targets with high nodal strength, including JUN, ROR2, COX-2, NF-κB, and FSP1 (Figure 6e), all of which have been experimentally validated in the present study (Figure 5).

#### 3.9.3. Gene Ontologies (GO) and Enriched Analysis of Quercetin Targets in AD

**Figure 7:**
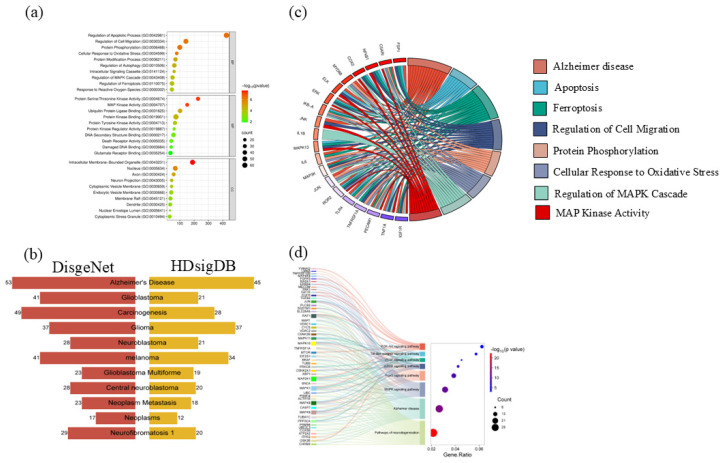
Functional annotation of Alzheimer’s disease (AD)-associated pathways. (**a**) Bubble plot showing the top 10 gene ontologies, including Biological Processes, Cellular Components, and Molecular Functions. (**b**) The two-way butterfly plot indicating the top 11 predicted diseases from DisgeNet and HDsigDB databases. (**c**) Chord diagram illustrating the top 8 gene ontologies and their corresponding AD-associated target. (**d**) Sankey plot depicting the top 8 signalling pathways and their shared genes targets.

Functional enrichment of quercetin interaction with AD-associated identified significant GO terms across Biological Processes (BP), Cellular Components (CC), and Molecular Functions (MF), highlighting apoptotic signalling, oxidative-stress response, and MAPK cascade regulation, with CC enrichment in membrane rafts, axons, nuclei, neuron projections, and dendrites (Figure 7a).

Disease-association analyses (DisGeNet and HDsigDB) ranked AD as the top hit, followed by other neurological disorders such as Parkinson’s disease, glioblastoma, and epilepsy, reflecting shared molecular mechanisms across neurodegenerative conditions (Figure 7b). The chord diagram (Figure 7c) mapped the top eight GO terms to specific target genes, including JUN, JNK, ROR2, COX-2, MAP kinase, and NF-κB, all highly enriched in AD-related pathways. Sankey plot visualisation (Figure 7d) further illustrated the strong interconnections between these pathways and their shared genes, linking apoptosis, ferroptosis, oxidative-stress regulation, and MAPK/NF-κB signalling.

#### 3.9.4. Binding Interaction of Quercetin with Target Genes Using Molecular Docking

**Figure 8:**
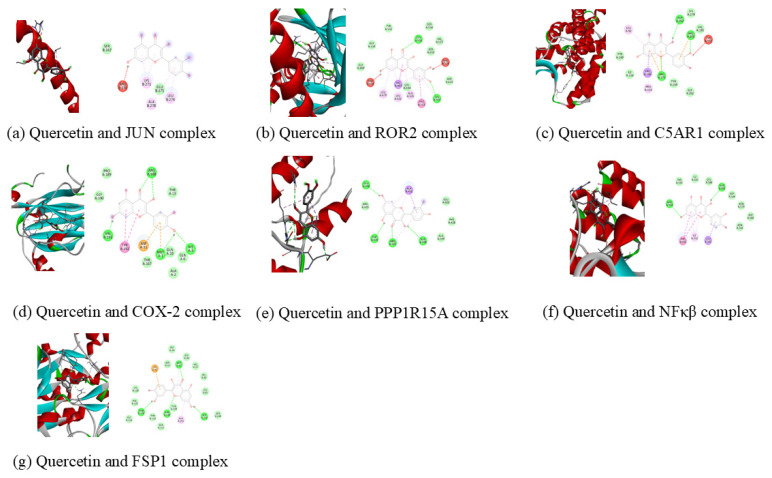
Molecular docking of quercetin, a major polyphenol present in sorghum, with AD-associated proteins (genes). (**a**) JUN (**b**) ROR2 (**c**) C5AR1 (**d**) COX-2 (**e**) PPP1R15A (**f**) NF-κB (**g**) FSP1. Molecular docking was performed using AutoDock 1.5.4; 2D/3D interactions visualised in BIOVIA Discovery Studio. JUN: c-Jun N-terminal kinase; ROR2: Receptor Tyrosine Kinase-Like Orphan Receptor 2; C5AR1: Complement Component 5a Receptor 1; COX- 2: Cyclooxygenase-2; PPP1R15A: Protein Phosphatase 1 Regulatory Subunit 15a; NFκβ: Nuclear Factor Kappa-Light-Chain-Enhancer of Activated B cells; FSP1: Ferroptosis Suppressor Protein 1.

Molecular docking was performed to evaluate interactions between quercetin and AD-associated target proteins (genes) identified earlier by experimental and bioinformatics analyses. Binding energies reflect strong quercetin–protein interactions (−5.0 to −9.7 kcal mol^−1^; Table S2). Docking analysis showed that quercetin strongly interact with AD-associated proteins (Figure 8), including ROR2, C5AR1, COX-2 (PTGS2), NF-κB, FSP1, and PPP1R15A, through hydrogen bonds and covalent interactions (Figure 8), consistent with the strong binding affinities reported in Table S2. Overall, these docking results support quercetin’s potential to modulate with multiple MAPK/NFκB signalling proteins, inflammatory mediators, and ferroptosis regulators.

## 4. Discussion

*Sorghum bicolor*, a polyphenol-rich grain, has attracted growing research interest as a potential complementary intervention for AD, mainly due to its abundant reserve of polyphenols including kaempferol, gallic acid, quercetin, and, notably, its uniquely high content of 3-deoxyanthocyanins, which collectively confer antioxidant, anticancer, antidiabetic, and gut microbiome modulating properties [31,32,33,34,35,36,37]. Building on our previous findings, crude polyphenol extracts (CPEs) and purified polyphenol extracts (PPEs) from three *Sorghum bicolor* genotypes (black (BS), red-brown (RB) and red (RS)) were selected for investigation based on their anti-Aβ_42_ aggregation and neuroprotective potential. Through an integrated approach combining biochemical assays, cell culture, transcriptomic and bioinformatics analyses, we comprehensively investigated their effects on AD pathologies using MC-65 cell line. The MC-65 cell line conditionally expresses the carboxyl-terminal 99-amino acid fragment of the amyloid precursor protein (APP-C99). It serves as high-throughput platform that provides valuable insights for early-stage drug discovery in AD research. Our results demonstrate that CPEs and PPEs, particularly from CBS and PBS, effectively inhibit Aβ_42_ aggregation, attenuate mitochondrial dysfunction, restore antioxidant enzyme activity, and modulate AD-associated genes and pathways. In silico analyses further revealed their effects were attributable to quercetin and other polyphenols present in the extracts.

The Thioflavin T assay showed that all sorghum polyphenol extracts significantly reduced Aβ_42_ fibril formation compared with the untreated Aβ_42_ control. The inhibitory effect was most pronounced for the PBS and CBS, both of which produced inhibition comparable to quercetin. Aggregation kinetic curves for these treatments showed an extended lag phase and reduced plateau, indicating both slower nucleation and lower overall fibril load. Endpoint fluorescence measurements corroborated these kinetic observations, highlighting strong anti-amyloidogenic activity of these extracts. These findings are consistent with previous reports that plant-derived polyphenols disrupt β-sheet assembly and stabilise non-toxic Aβ intermediates [8]. The polyphenol composition of all six samples’ extracts has been comprehensively characterised using LC-HRMS and the detailed phytochemical profiling manuscript is currently under peer review for publication; relevant compositional data are incorporated in the Supplementary File. Across the extracts, PPEs inhibited Aβ_42_ aggregation more effectively than CPEs, likely due to the higher concentration and bioactivity achieved after purification using ethyl acetate. PRS displayed weaker inhibition at lower concentrations, possibly reflecting differences in its polyphenol composition.

Western blot analysis corroborated the ThT assay results, demonstrating that polyphenol treatments reduced the accumulation of soluble Aβ_42_ oligomers—the most neurotoxic Aβ species in AD. The reduction correlated with improved cell viability, suggesting that reducing toxic intermediates is a major driver of the observed neuroprotection. These results indicate that quercetin-rich sorghum polyphenol extracts, particularly PPEs, disrupt Aβ_42_ aggregation process, from early nucleation to fibril elongation, thereby reducing the formation of toxic oligomers and preserving neuronal health.

Exposure to Aβ_42_ caused marked disruption of mitochondrial networks in MC-65 cells, characterised by loss of structural integrity and reduced mitochondrial mass. MitoTracker imaging revealed that untreated cells displayed fragmented mitochondria, indicative of mitochondrial stress; whereas, treatment with sorghum polyphenol extracts, especially the PBS, preserved the elongated, interconnected mitochondrial morphology typical of healthy cells. Quantitative fluorescence analysis confirmed significant recovery of mitochondrial activity in treated groups, with PBS showing near-complete restoration to Tet. control levels. These structural improvements were supported by ATP assay, which demonstrated that CBS, PBS, PRS, and quercetin reversed Aβ-induced ATP depletion at 250–500 µg/mL, implying restoration of mitochondria bioenergetic function. The mitochondrial benefits observed here are consistent with our previous findings, where polyphenol extracts from six sorghum varieties increased ATP levels in BE-17 cells by 25 – 38% (*p* < 0.01) [8], and with reports of similar neuroprotective effects from grape seed extract, which elevated intracellular ATP in Aβ_42_-induced SH-SY5Y cells by over 100% within 24 h [38].

Oxidative stress is a major driver of AD pathology and often precedes Aβ senile plaques formation. In our study, all three sorghum extracts, particularly CBS and PBS, significantly reduced ROS and enhanced the activity of catalase and peroxidase. The significant elevation in peroxidase activity in CBS- and PBS-treated cells suggests a fortified endogenous antioxidant defence capable of disrupting the cycle of oxidative damage and Aβ-induced toxicity. These findings are consistent with reports on curcumin, which reduces oxidative stress, upregulates antioxidant enzymes, and modulates protective signalling pathways across multiple AD models [39,40,41].

A major finding of this study is the elucidation of mechanisms underlying the neuroprotective functions of quercetin-rich sorghum polyphenol extracts (CBS, PBS, PRS) and quercetin. RNA-sequencing revealed that intracellular accumulation of Aβ_42_ strongly upregulated MAPK/NF-κB-associated inflammatory genes (JUN, ROR2, COX-2, PPP1R15A, NFκB1) while downregulating the ferroptosis-suppressing protein (FSP1). Treatment with 250 µg/mL CBS, PBS, or PRS significantly reversed these changes, restoring a transcriptional profile consistent with reduced neuroinflammation and improved ferroptosis resistance. MAPK activation is known to increase NF-κB activity, which drives the expression of pro-inflammatory mediators such as iNOS, COX-2, and interleukins, changes frequently observed in AD brain tissue [42,43], and is also associated with Aβ accumulation, tau phosphorylation, synaptic dysfunction, and cognitive impairment [29]. Our results align with previous works showing that polyphenols (such as resveratrol analogues, curcumin, luteolin, and apigenin) suppress MAPK/NF-κB signalling and downregulate inflammatory gene expression in neuronal and glial models [44,45,46]. Importantly, sorghum polyphenol extracts significantly upregulated FSP1, a protein that inhibits ferroptosis by regenerating reduced coenzyme Q_10_, α-tocopherol, and vitamin K, thereby neutralising lipid peroxyl and oxyradicals [47,48]. While reports on polyphenols modulating FSP1 are scarce, our data suggest this pathway may be an important, previously underreported mechanism of neuroprotection.

Analysis of the public dataset (GSE5281) confirmed that several MAPK/NF-κB- and ferroptosis-related targets identified in our study are dysregulated in human AD brain tissue. Bioinformatics analyses identified quercetin as a polyphenol linking *S. bicolor* metabolites to AD-associated pathways, pinpointing 15 hub genes involved in apoptosis regulation, oxidative stress response, MAPK signalling, and ferroptosis. Molecular docking supported these findings, demonstrating strong quercetin binding to multiple targets, including ROR2, FSP1, ERK, C5AR1, JUN, PPP1R15A, NF-κB, and COX-2. Integrating the docking with the RNA-sequencing results reinforces the conclusion that quercetin (and other major polyphenol in sorghum grain) exerts neuroprotection through simultaneous modulation of inflammation, oxidative stress, mitochondrial integrity, and Aβ_42_ aggregation inhibition, all pathological processes in AD. Collectively, these findings suggest that CBS, PBS, and PRS may inhibit AD progression by suppressing MAPK/NF-κB activation and ferroptosis.

Taken together, these findings highlight the therapeutic potential of *Sorghum bicolor* polyphenol extracts in targeting multiple pathological hallmarks of AD. PPEs show stronger activity than CPEs; yet, the crude extracts retained substantial bioactivity, indicating that optimised extraction and formulation could further enhance their efficacy. The distinct, genotype-specific effects observed across CBS, PBS, and PRS emphasise the importance of comprehensive phytochemical profiling to identify the most potent neuroprotective polyphenols. Although these results are promising, they were obtained in a single cell model under controlled in vitro conditions. More robust mechanistic investigations—including organoids and in vivo AD models—are required to elucidate how these extracts influence Aβ clearance, oxidative stress regulation, and mitochondrial function. Advancing this knowledge will be essential for the development of *Sorghum bicolor* polyphenols as effective, evidence-based complementary therapies for Alzheimer’s disease.

## 5. Conclusions

Integrating the docking findings with RNA sequencing results strengthens the conclusion that quercetin-rich sorghum polyphenol extracts exert neuroprotective effects through multitarget modulation, simultaneously impacting inflammation, oxidative stress, mitochondrial integrity, and protein aggregation. Collectively, these findings speculates that CBS, PBS, and PRS may inhibit AD progression by suppressing MAPK/NF-κB activation and ferroptosis while enhancing neuronal resilience to Aβ-induced toxicity.

## Data Availability

All data generated or analysed during this study are included in this published article and its Supplementary Information files.

## Supplementary Materials

The following supporting information can be downloaded at: https://www.mdpi.com/article/10.3390/nu18132121/s1, Figure S1: Expression analysis of Alzheimer’s disease gene targets retrieved from GEO omnibus data sets GSE5281; Table S1: Toxicity of higher concentrations of the sorghum polyphenol extracts on MC-65 cells; Table S2: Molecular docking results of various genes implicated in Alzheimer’s disease.

## Abbreviations

The following abbreviations are used in this manuscript:

AD	Alzheimer’s disease
ATP	Adenosine triphosphate
AU	Arbitrary units
Aβ	Amyloid beta
BP	Biological Processes
C5AR1	Complement component 5a receptor 1
CAT	Catalase
CBS	Crude black sorghum
CC	Cellular Components
COX-2	Cyclooxygenase-2
CPEs	Crude polyphenol extracts
CRB	Crude red-brown sorghum
CRS	Crude red sorghum
CTD	Comparative Toxicogenomics Database
DMEM	Dulbecco’s modified eagle medium
DMSO	Dimethyl sulfoxide
FBS	Foetal Bovine serum
FSP1	Ferroptosis suppressor protein 1
GEO	Gene Expression Omnibus
GO	Gene ontology
HBSS	Hank’s balanced salt solution
HFIP	1,1,1,3,3,3-Hexafluoro-2-propanol
JUN	c-Jun N-terminal kinase
KEGG	Kyoto Encyclopaedia of Genes and Genomes
MAPK	Mitogen-activated protein kinase
MF	Molecular Functions
MTS	3-(4,5-dimethylthiazol-2-yl)-5-(3-carboxymethoxyphenyl)-2-(4-sulfophenyl)-2H-tetrazolium)
NF-κB	Nuclear Factor Kappa-Light-Chain-Enhancer of Activated B cells
PBS	Purified black sorghum
PDB	Protein Data Bank
PER	Peroxidase
PPEs	Purified polyphenol extracts
PPP1R15A	Protein phosphatase 1 regulatory subunit 15A
PRB	Purified red-brown sorghum
PRS	Purified red sorghum
ROR2	Receptor tyrosine kinase-like orphan receptor 2
ROS	Reactive oxygen species
RT	Room temperature
STITCH	Search tools for interacting chemicals
TBS	Tris-buffered saline
Tet	Tetracycline
Th-T	Thioflavin T
