# Supplementary material for: Neuroprotective Effects of Sorghum Polyphenol in Alzheimer’s Disease: In Vitro and In Silico Analyses
Source: Nutrients. 2026 Jun 30;18(13):2121. doi: 10.3390/nu18132121 (PMC13364096; doi:10.3390/nu18132121)
Supplement: Supplementary file 1 [file nutrients-18-02121-s001.zip › Supplementary Figure.pdf]

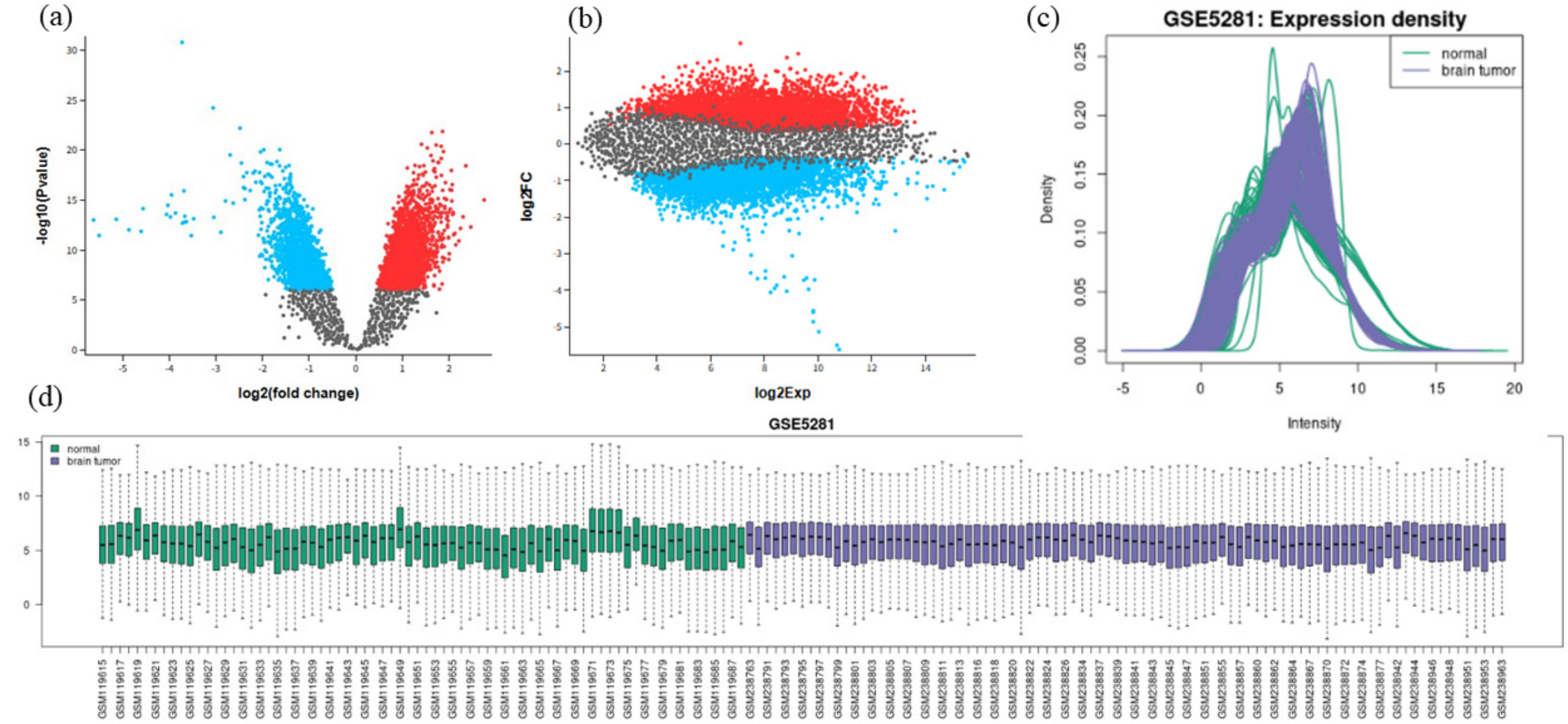

Figure S1. Expression analysis of Alzheimer's disease (AD) gene targets retrieved from GEO omnibus data sets GSE5281. (a,b) Volcano plots for GSE5281 datasets represents the upregulated genes (red dots) and down-regulated (blue dots), and no statistically significantly difference (block dots). (c) Expression density of target genes in normal and AD brain. (d) GSE database showing expression levels represented in TPM. Green colour box plot represents normal and purple colour box plot indicate AD samples.
