# Supplementary material for: Neuroprotective Effects of Sorghum Polyphenol in Alzheimer’s Disease: In Vitro and In Silico Analyses
Source: Nutrients. 2026 Jun 30;18(13):2121. doi: 10.3390/nu18132121 (PMC13364096; doi:10.3390/nu18132121)
Supplement: Supplementary file 1 [file nutrients-18-02121-s001.zip › Supplementary Tables.pdf]

Supplementary Table

Table S1 Toxicity of higher concentrations of the sorghum polyphenol extracts on MC-65 cells

Treatment	%Viability
750 µg/mL- CBS	22.12 ± 21
1000 µg/mL- CBS	18.10 ± 16
750 µg/mL- CRB	24.81 ± 11
1000 µg/mL- CRB	19.10 ± 18
750 µg/mL- CRS	28.15 ± 51
1000 µg/mL- CRS	20.32 ± 88
750 µg/mL- PBS	23.54 ± 17
1000 µg/mL- PBS	21.28 ± 81
750 µg/mL- PRB	26.02 ± 34
1000 µg/mL- PRB	20.50 ± 14
750 µg/mL- PRS	26.45 ± 22
1000 µg/mL- PRS	25.23 ± 65

CBS: crude black sorghum, CRB: crude red-brown, CRS: crude red sorghum, PBS: purified black sorghum, PRB: purified red-brown, PRS: purified red sorghum.

Table S2 Molecular docking results of various genes implicated in Alzheimer's disease.

Proteins	Binding energy (kcal/mol)	Number of hydrogen bonds	Residues
C5ARI	-7.6	3	ASP A:282, ARG A:206, ARG A:175
COX-2	-6.5	4	ARG A:188, VAL A:191, MET A:1, HIS A:3
ELK	-6.4	2	ASP A:30, LEU A:15
ERK	-8.5	3	MET A:125, ASP A:128, GLN A:122
FSP-1	-9.4	4	ASN A:120, ASN A:248, MET A:42,SER A:250
IKB-A	-9.7	3	THR A:23, GLU A:97,CYS A:99
IL-1B	-7.2	6	LYS A:254, THR A:260,GLY A:255 GLN A:257, GLN A:130,ASN A:223
IL-6	-7.7	2	ARG A:196, LEU A:90
JNK	-8.3	-	-
MAP-3K	-8	3	VAL A:729, LEU A:658,ASP A:777
MAPK-13	-7.9	1	HIS A:142
MYD-88	-6.5	3	GLN A:249,THR A:272,CYS A:274
NF-kB	-6.7	2	ARG A:934, GLN A:583
PPP1R115A	-6.5	4	GLU A:438, ASP A:439,ARG A:595 GLN A:598
JUN	-5	-	-
ROR-2	-9.5	2	CYS A:556, LYS A:507
TLR-4	-8	2	LEU A:815, ASP A:675
TNF-A	-7	4	PRO A:215, LEU A:218,LYS A:141, PHE A:220
